# Supplementary material for: Identification of Skin Lesions by Snapshot Hyperspectral Imaging
Source: Cancers (Basel). 2024 Jan 2;16(1):217. doi: 10.3390/cancers16010217 (PMC10778186; doi:10.3390/cancers16010217)
Supplement: Supplementary file 1 [file cancers-16-00217-s001.zip › cancers-2781525-supplementary.pdf]

cancers

Supplementary Materials

Identification of Skin Lesions by Snapshot Hyperspectral Imaging

Hung-Yi Huang [1], Hong-Thai Nguyen [2], Teng-Li Lin [3], Penchun Saenprasarn [4], Ping-Hung Liu [5,*] and Hsiang-Chen Wang [2,6,*]

1 Department of Dermatology, Ditmanson Medical Foundation Chiayi Christian Hospital, Chia Yi City 60002, Taiwan; 07117@cych.org.tw
2 Department of Mechanical Engineering, National Chung Cheng University, 168, University Rd., Min Hsiung, Chia Yi 62102, Taiwan
3 Department of Dermatology, Dalin Tzu Chi General Hospital, No. 2, Min-Sheng Rd., Dalin Town, Chiayi 62247, Taiwan; tanglilin1121@hotmail.com
4 School of Nursing, Shinawatra University, 99 Moo 10, Bangtoey, Samkhok, Pathum Thani 12160, Thailand; pencbun_sa@mru.ac.th
5 Division of General Surgery, Department of Surgery, Kaohsiung Armed Forces General Hospital, 2, Zhongzheng 1st.Rd., Lingya District, Kaohsiung City 80284, Taiwan
6 Director of Technology Development, Hitspectra Intelligent Technology Co., Ltd., Kaohsiung 80661, Taiwan
* Correspondence: a0968020005@mail.802.org.tw (P.-H.L.); hcwang@ccu.edu.tw (H.-C.W.)

S1. Hyperspectral Imaging Technology

S1.1. XYZ Color Matching Function

Color matching functions (CMFs) are set of three functions that represent the average response of the human eye to light of different wavelengths. These functions are derived from experimental observations and are central to the theory of the CIE (International Commission on Illumination) color spaces.

$\bar{x}(\lambda)$: Represents the response of the eye to red light, $\bar{y}(\lambda)$: Represents the response to green light and also corresponds to the luminosity function (which describes how bright a light appears), $\bar{z}(\lambda)$: Represents the response to blue light.

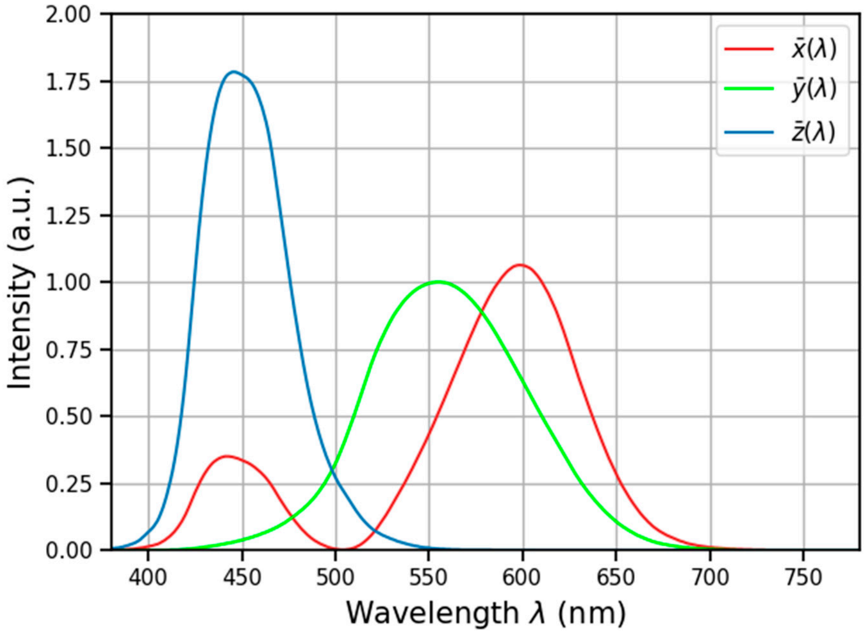

Figure S1. XYZ color matching function.

S1.2. Linearize (Remove Gamma Correction)

For sRGB, the gamma correction is approximately a power function with a power of 2.2, but with a linear segment for small values. The formula for gamma correction removal (linearization) is:

$$f(n) = \begin{cases} \left(\frac{n+0.055}{1.055}\right)^{2.4}, & n > 0.04045 \\ \left(\frac{n}{12.92}\right), & otherwise \end{cases} \tag{S1}$$

where n is the color value in sRGB space.

S1.3. sRGB to XYZ Conversion

This equation facilitates the conversion of color values from the sRGB color space to the standard XYZ color space, accommodating the specific XYZ parameters of the camera utilized, contingent upon the lighting conditions of the measurement environment. Subsequently, linear RGB values are transformed into the XYZ color space using a designated transformation matrix, as delineated in Equation (S2).

$$\begin{bmatrix} X \\ Y \\ Z \end{bmatrix} = [M_A][T] \begin{bmatrix} f(R_{sRGB}) \\ f(G_{sRGB}) \\ f(B_{sRGB}) \end{bmatrix} \times 100, 0 \leqq \begin{matrix} R_{sRGB} \\ G_{sRGB} \\ B_{sRGB} \end{matrix} \leqq 1 \tag{S2}$$

where $[T]$ is the transformation matrix, defined as follow:

$$[T] = \begin{bmatrix} 0.4104 & 0.3576 & 01805 \\ 0.2126 & 0.7152 & 0.0722 \\ 0.0193 & 0.1192 & 0.9505 \end{bmatrix} \tag{S3}$$

However, during the conversion, because the sRGB color gamut space defines the White point as the D65 light source (X_{CW}, Y_{CW}, Z_{CW}) rather than the white point of the measurement light source (X_{SW}, Y_{SW}, Z_{SW}), so we adapt the conversion matrix through the color M_A is used to convert the white point of the D65 light source into the white point of the measurement light source, as shown in Equation (S4).

$$[M_A] = \begin{bmatrix} X_{SW}/X_{CW} & 0 & 0 \\ 0 & Y_{SW}/Y_{CW} & 0 \\ 0 & 0 & Z_{SW}/Z_{CW} \end{bmatrix} \tag{S4}$$

*S1.4. XYZ to L*a*b* Color Space Conversion*

In this investigation, the wavelength band employed encompasses a portion of the visible spectrum, ranging from 380 nm to 780 nm. Consequently, the outcomes of the correction can be appropriately expressed in terms of chromatic aberration. To quantify this chromatic aberration, the CIEDE 2000 standard is utilized, which offers a comprehensive approach by incorporating hue rotation, neutral color compensation, luminance compensation, chromaticity compensation, and tone compensation. These adjustments effectively address the human eye's varying sensitivity to different colors. Prior to the application of the CIEDE 2000 formula, it is imperative to convert the color values from $XYZ_{correction}$ and $XYZ_{spectrometer}$ to the Lab color space.

First, the XYZ values are normalized against a reference white point. The reference white is a theoretically perfect diffuser and is defined in terms of XYZ values. Common reference whites include D65 (daylight), D50 (midday light), and others. Let's denote the normalized values as X_n, Y_n, and Z_n, where $X_n=X/X_{ref}$, $Y_n=Y/Y_{ref}$, *and* $Z_n=Z/Z_{ref}$.

The conversion from XYZ to Lab color space involves a series of calculations that map the XYZ color values to the CIELAB color space, which is designed to be more

perceptually uniform. The CIELAB color space consists of three values: L^* (lightness), a^* (green-red), and $b*$ (blue-yellow). This conversion process is detailed in Equations (S5)–(S8).

$$L^* = 116f\left(\frac{Y}{Y_n}\right) - 16 \tag{S5}$$

$$a^* = 500\left[f\left(\frac{X}{X_n}\right) - f\left(\frac{Y}{Y_n}\right)\right] \tag{S6}$$

$$b^* = 500\left[f\left(\frac{X}{X_n}\right) - f\left(\frac{Y}{Y_n}\right)\right] \tag{S7}$$

where the function *f(n)* is defined as:

$$f(n) = \begin{cases} n^{\frac{1}{3}}, n > 0.008856 \\ 7.787n + 0.137931, otherwise \end{cases} \tag{S8}$$

S1.5. CIEDE 2000 Color Difference

The CIEDE2000 color difference formula is complex, involving several intermediate calculations and adjustments. Here is the general formula for the color difference, ΔE_{00}, according to CIEDE2000:

$$\Delta E_{00} = \sqrt{\left(\frac{\Delta L'}{k_L S_L}\right)^2 + \left(\frac{\Delta C'}{k_C S_C}\right)^2 + \left(\frac{\Delta H'}{k_H S_H}\right)^2 + R_T \frac{\Delta C'}{k_C S_C}\frac{\Delta H'}{k_H S_H}} \tag{S9}$$

where:

- $\Delta L'$, $\Delta C'$, and $\Delta H'$ are the differences in lightness, chroma, and hue between the two colors, respectively, modified by the conditions of the observation (like illumination).
- S_L, S_C, and S_H are the scaling factors for lightness, chroma, and hue, respectively.
- R_T is the rotation function accounting for the interaction between chroma and hue in the blue region.
- k_L, k_C, and k_H are parametric factors usually set to 1 in typical viewing conditions.

The calculation of $\Delta L'$, $\Delta C'$, and $\Delta H'$ involves several steps, starting with the computation of the CIELAB values (L^*, a^*, b^*) for each color, then computing their respective C^* (chroma) and $\hbar$ (hue) values, and then making adjustments to these based on the specific colors being compared.

The CIEDE2000 formula is significantly more accurate in representing human perception of color differences compared to its predecessors (like CIELAB and CIE94), especially for small color differences and in problematic hue areas such as blues and greens. However, due to its complexity, it's often implemented through software in practical applications.

Table S1. Root Mean Square Error of $S_{spectrum}$ and $R_{spectrometer}$.

color no.	RMSE	MSE	MAE
color 1	0.0641	0.0041	0.0357
color 2	0.0537	0.0029	0.0432
color 3	0.0675	0.0046	0.0482
color 4	0.0783	0.0061	0.0416
color 5	0.0670	0.0045	0.0441
color 6	0.0474	0.0022	0.0330
color 7	0.0833	0.0069	0.0669
color 8	0.0253	0.0006	0.0199
color 9	0.0677	0.0046	0.0496
color 10	0.1121	0.0126	0.0725
color 11	0.0203	0.0004	0.0157
color 12	0.0482	0.0023	0.0405
color 13*	0.0346	0.0012	0.0296
color 14*	0.0259	0.0007	0.0193
color 15*	0.0891	0.0079	0.0678
color 16*	0.0526	0.0028	0.0431
color 17*	0.0628	0.0039	0.0495
color 18*	0.0345	0.0012	0.0301
color 19	0.0055	0.0000	0.0044
color 20	0.0320	0.0010	0.0248
color 21	0.0667	0.0044	0.0463
color 22	0.0648	0.0042	0.0432
color 23	0.0422	0.0018	0.0278
color 24	0.0155	0.0002	0.0105
average error	0.0525	0.0034	0.0378

S1.6. Third Order Linear Regression Analysis

The coefficient of determination, denoted as R^2, is a statistical measure that represents the proportion of the variance for the dependent variable that's explained by independent variables in a regression model. It provides a measure of how well observed outcomes are replicated by the model, based on the proportion of total variation of outcomes explained by the model.

For simple linear regression, R^2 is the square of the Pearson correlation coefficient (often denoted r). It can range from 0 to 1:

- An R^2 of 1 indicates that the regression predictions perfectly fit the data.
- An R^2 of 0 indicates that the model does not explain any of the variance in the response variable.

The formula for R^2 is defined as:

$$R^2 = 1 - Sum\ of\ squares\ of\ residuals\ (SSR)/Total\ sum\ of\ squares\ (SST)$$

where

- SSR is the sum of the squares of the difference between the observed and predicted values.
- SST is the sum of the squares of the difference between the observed values and the mean of the observed values.

S1.7. Principal Component Analysis of Reflectance Spectrum

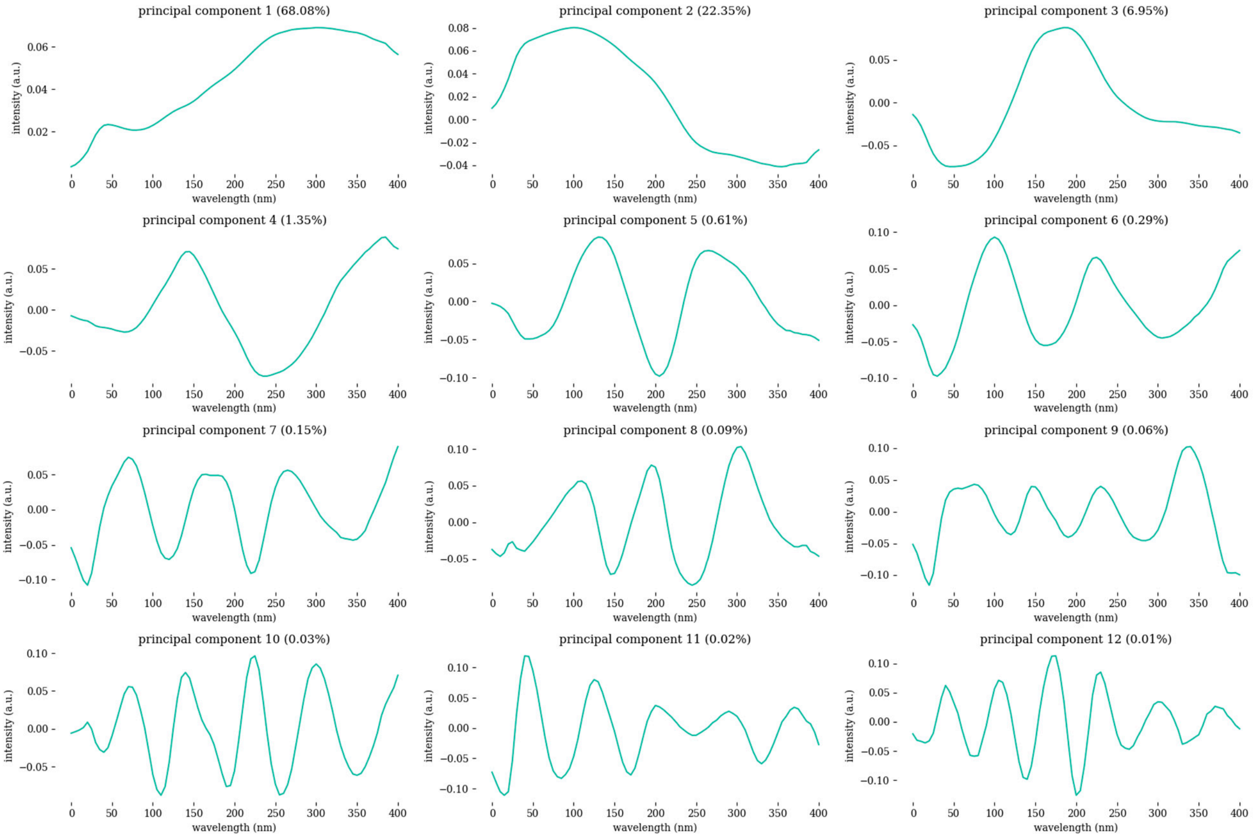

Figure S2. 12 principal components of $R_{spectrometer}$.

S2. Evaluation Metrics

In deep learning, the confusion matrix (Confusion matrix) is often used as an evaluation. The confusion matrix integrates the predicted classification and the actual classification, and then separates True positive (TP): The number of correctly predicted positive cases; False positive (FP): The number of actual negative cases incorrectly predicted as positive; False negative (FN): The number of actual positive cases incorrectly predicted as negative; and True Negative (TN): The number of correctly predicted negative cases; four categories according to these four categories to calculate the indicators, including Sensitivity, Specificity and F1-score.

Sensitivity measures the proportion of true positive predictions (correctly identified positive cases) out of all actual positive cases.

$$Sensitivity = \frac{TP}{TP + FN}$$

Precision refers to the actual accuracy when the prediction is positive. This indicator can be used to identify whether the predicted result is meaningful, as shown in Formula (3-2).

$$Precision = \frac{TP}{TP + FP}$$

Specificity measures the proportion of true negative predictions (correctly identified negative cases) out of all actual negative cases.

$$Specificity = \frac{TN}{FP + TN}$$

F1-Score is the harmonic mean of precision and sensitivity. It provides a balance between precision (positive predictive value) and recall (sensitivity).

$$F1 - score = \frac{2}{\frac{1}{Precision} + \frac{1}{Recall}}$$

ROC-AUC (Receiver Operating Characteristics - Area Under the Curve):

- ROC-AUC measures the area under the Receiver Operating Characteristic curve, which represents the true positive rate (sensitivity) versus the false positive rate (1 - specificity) at different threshold values.
- ROC-AUC quantifies a model's overall ability to distinguish between positive and negative cases, regardless of the chosen threshold.

The perfect classifier has an ROC-AUC of 1, while the random classifier has an ROC-AUC of 0.5.

Disclaimer/Publisher's Note: The statements, opinions and data contained in all publications are solely those of the individual author(s) and contributor(s) and not of MDPI and/or the editor(s). MDPI and/or the editor(s) disclaim responsibility for any injury to people or property resulting from any ideas, methods, instructions or products referred to in the content.
